# Sexual Dimorphism and Population Differences in Structural Properties of Barn Swallow (*Hirundo rustica*) Wing and Tail Feathers

**DOI:** 10.1371/journal.pone.0130844

**Published:** 2015-06-25

**Authors:** Péter L. Pap, Gergely Osváth, José Miguel Aparicio, Lőrinc Bărbos, Piotr Matyjasiak, Diego Rubolini, Nicola Saino, Csongor I. Vágási, Orsolya Vincze, Anders Pape Møller

**Affiliations:** 1 Evolutionary Ecology Group, Hungarian Department of Biology and Ecology, Babeş-Bolyai University, Cluj Napoca, Romania; 2 MTA-DE “Lendület” Behavioural Ecology Research Group, Department of Evolutionary Zoology and Human Biology, University of Debrecen, Debrecen, Hungary; 3 Museum of Zoology, Babeş-Bolyai University, Cluj Napoca, Romania; 4 Grupo de Investigación de la Biodiversidad Genética y Cultural, IREC-(CSIC-UCLM-JCCM), Ciudad Real, Spain; 5 ‘Milvus Group’ Bird and Nature Protection Association, Tîrgu Mureș, Romania; 6 Faculty of Biology and Environmental Sciences, Cardinal Stefan Wyszyński University in Warsaw, Warsaw, Poland; 7 Dipartimento di Bioscienze, Università degli Studi di Milano, Milano, Italy; 8 Laboratoire d’Ecologie, Systématique et Evolution, CNRS UMR 8079, Université Paris-Sud, Orsay Cedex, France; University of Lausanne, SWITZERLAND

## Abstract

Sexual selection and aerodynamic forces affecting structural properties of the flight feathers of birds are poorly understood. Here, we compared the structural features of the innermost primary wing feather (P1) and the sexually dimorphic outermost (Ta6) and monomorphic second outermost (Ta5) tail feathers of barn swallows (*Hirundo rustica*) from a Romanian population to investigate how sexual selection and resistance to aerodynamic forces affect structural differences among these feathers. Furthermore, we compared structural properties of Ta6 of barn swallows from six European populations. Finally, we determined the relationship between feather growth bars width (GBW) and the structural properties of tail feathers. The structure of P1 indicates strong resistance against aerodynamic forces, while the narrow rachis, low vane density and low bending stiffness of tail feathers suggest reduced resistance against airflow. The highly elongated Ta6 is characterized by structural modifications such as large rachis width and increased barbule density in relation to the less elongated Ta5, which can be explained by increased length and/or high aerodynamic forces acting at the leading tail edge. However, these changes in Ta6 structure do not allow for full compensation of elongation, as reflected by the reduced bending stiffness of Ta6. Ta6 elongation in males resulted in feathers with reduced resistance, as shown by the low barb density and reduced bending stiffness compared to females. The inconsistency in sexual dimorphism and in change in quality traits of Ta6 among six European populations shows that multiple factors may contribute to shaping population differences. In general, the difference in quality traits between tail feathers cannot be explained by the GBW of feathers. Our results show that the material and structural properties of wing and tail feathers of barn swallows change as a result of aerodynamic forces and sexual selection, although the result of these changes can be contrasting.

## Introduction

Morphology of wing and tail feathers of birds may vary depending on aerodynamic forces to which they are subject and on their function in intra- and inter-sexual signaling [1–6]. Wing feathers are exposed to strong aerodynamic forces during flight, and, therefore, flight style might determine the structure of these feathers [6,7]. Tail feathers, on the other hand, are subjected to reduced aerodynamic forces due to their almost exclusive role in aiding maneuverability, improving stability and controlling pitching and their limited role in generating lift [8–12]. Therefore, birds might invest less in structure and mechanical resistance of tail relative to wing feathers. However, with the exception of a few observations, little empirical research has addressed the functional morphology of different flight feather types (i.e. remiges and rectrices) in birds (but see [4,5,10,13]).

In some bird species, males have evolved exaggeratedly long tails as a result of sexual and natural selection [1,14–16], and the difference between the sexes in tail length may differ between populations [17]. Evolutionary elongation of tails should occur at the expense of structural complexity [2]. The predicted consequence of the difference between sexes in tail length is sexual dimorphism in the structural components of these feathers. If such sex-dependence is driven by dimorphism in tail length, then the difference between males and females in tail length may solely explain the simpler structure of long rectrices. In this case, tail elongation and the associated changes in structural components may ultimately lead to a differential cost of bearing these traits due to higher frequency of breakage of the elongated feathers [1,18,19]. On the other hand, if the sexual difference in feather traits persists even after controlling for dimorphism, then sex related variation in tail length does not solely explain the sexual dimorphism in feather structure. This may show that males do not fully compensate for elongation of the tail.

Feathers consist of a central shaft called the rachis. Barbs are attached sub-perpendicularly to the rachis and carry the barbules. Feather vanes are formed by parallel barbs that are connected via hook and bow barbules, and are light, flexible, and resistant to damage [20]. The amount of resources invested in feather production is higher and the feather has superior structural quality (i.e. larger rachis width, more dense barbs- and barbules) when food resources are abundant, healthy and slow-molting birds [21–26]. Furthermore, the flexural stiffness of flight feathers, which reflects the mechanical properties of the rachis, is reduced in birds with fast molt and large parasite load [21,23,24]. Finally, a comparative study of European birds has shown that species with more active flight (i.e. with higher aerodynamic forces acting on the wing) and with prolonged molt have higher barb density on wing feathers [6]. These findings show that rachis width, barb and barbule density of the vane, and flexural stiffness are good indicators of feather quality and the aerodynamic forces to which they are exposed.

The barn swallow (*Hirundo rustica*) is an aerially feeding passerine species that exhibits sexually dimorphic outer tail feathers (i.e. streamers). Sexual dimorphism may vary among different European populations according to the strength of natural and sexual selection to which they are subject [1,16,17]. Wing feathers, however, are likely to be shaped largely by natural selection, because their primary function is to generate lift and thrust during flight. These characteristics make the barn swallow an ideal model organism for studies of the evolution of feather structure. In order to determine the change in structural elements of flight feathers in relation to aerodynamic forces, we compared the features of the innermost primary feather (henceforth P1) with the outermost and the second outermost tail feathers (henceforth Ta6 and Ta5, respectively) collected from a Romanian breeding population. Because tail feathers are exposed to a lower aerodynamic force as compared to P1, we expected the structural elements of the P1 to be stronger (larger rachis width, higher barb- and barbule density, higher bending stiffness) than those of tail feathers. Furthermore, because Ta6 is elongated, we expected the structural elements of this feather to be modified in males compared to females. However, no sexual difference in feather quality was expected for P1 and Ta5, which shows a lower sex dimorphism in length [1]. If the difference between sexes in quality of tail feathers is solely related to dimorphism in length, the difference between sexes should vanish after controlling for feather length. However, if males compensate for the increase in tail length, the difference between sexes in quality measures should persist even after controlling for feather length. Because the structural elements of feathers develop during growth, and feather growth rate determines feather quality (e.g. [21,26]), we further analyzed the relationship between quality measures and feather growth rate. We also tested if sexual dimorphism in feather quality of Ta6 in six geographically distinct barn swallow populations varied in relation to the degree of sexual dimorphism in tail length. Finally, we tested if the differences among populations in Ta6 growth rate might explain population differences in feather quality traits.

## Materials and Methods

### Field Data Collection

In 2013 we collected 124 P1, 107 Ta6 and 124 Ta5 from a Romanian breeding barn swallow population (Cojocna village, 46° 75’ N, 23° 84’ E, central Transylvania). In addition, we collected Ta6 feathers from five other barn swallow populations as well (Denmark: 57° 10’ N, 10° 00’ E (*N* = 18); Ukraine: 50° 21’ N, 30° 54’ E (*N* = 19); Poland: 52° 22’ N, 20° 53’ E (*N* = 60); Italy: 45° 33’ N, 8° 33’ E (*N* = 30); Spain: 39° 35’ N, 3° 40’ W (*N* = 62)). Feather samples were collected during the breeding season 2013, except the Spanish population where samples were collected in 2008.

We used mist nets and nest traps for capturing adult birds during the breeding season. Each adult barn swallow was ringed and standard biometrical traits were measured. We plucked P1 of the left wing and the left Ta5 from birds belonging to the Romanian population, and one of Ta6 from all six populations. We collected only fully grown feathers without any sign of damage. Sex was determined by the presence or absence of a brood patch, which develops in all females during the breeding season, but not in males ([1]; pers. obs.). The sex ratio in our samples was balanced in all six populations.

### Ethics Statement

The feathers were removed by gently pulling the feather from the distal end. All individuals were released as soon as possible, usually within 10 minutes of capture. After being released, swallows behaved normally and our observations of individuals confirmed that they resumed their normal breeding activities. The survival rate (i.e. re-captured in 2014 at the same locality) of birds from the Romanian population with (P1, Ta6 and Ta5) and without feathers removed was similar (*χ* ^2^ = 0.06, df = 121, *P* = 0.80). In addition, barn swallows commonly loose parts or all of their rectrices under natural conditions. The farmers gave permission to enter into their properties. This research was carried out in strict accordance with the national legislation and approved by the national agencies from Romania (Romanian Ornithological Centre, license no: 726965), Denmark (3446–00035), Ukraine (396-i.21.05.2012), Italy (Provincia di Novara, auth. n. 4309/2011), Poland (Polish Ornithological Station license no 211/2013) and Spain (authorization provided by the Spanish population Environmental Agency of the regional Government of Castilla-La Mancha (JCCM) for capturing and manipulating barn swallows). The research was further approved by an institutional animal care and use committee for the Danish and Ukrainian studies. The sampling procedures, due to the minimal effect on sampled subjects, did not require additional authorization from ethical boards for the Romanian, Italian, Spanish and Polish populations.

### Feather Trait Measurements

For each feather we measured total length, rachis width, barb and barbule density and bending stiffness. Feather length was measured with a ruler to the nearest 0.5 mm. Rachis width was measured across the dorso-ventral plane with a digital caliper to the nearest 0.01 mm at the base of the vane. We took digital photographs of the feathers laying on a metric grid background or a stage micrometer that was imported into ImageJ version 1.37 (http://rsb.info.nih.gov/ij/) to allow measurements of barb and barbule density, respectively. Barb and barbule density were measured on the inner feather vane near the midpoint of the rachis. The density of barbs and barbules was calculated as the number of embranchments of barbs and proximal barbules in the middle of the vane along 1 cm of rachis and a 1 mm barb length section, respectively. The stiffness of all feathers was measured using the method described by Dawson et al. [21]. A weight of two grams was attached to the rachis at two-third along its length measured from the proximal end of the feather, and the vertical (downward) deflection was measured at the distal end. The calamus was fixed into a hole between two thick rubber bands so that it emerged at approximately 10 mm from the proximal end. The holder was mounted on a tripod in front of a metric grid, with the dorsal surface of the feather pointing upwards. The weight was attached to the rachis by means of a short piece of cotton thread. Vertical deflection of the feather was measured from digital pictures taken before and after the weight attached and was expressed as the distance between the two measures. Higher deflection values thus indicate lower stiffness (for the confounding effect of the length see below).

We measured feather growth rate as growth bars width (GBW) on Ta5 (from the Romanian population) and on Ta6 only (from all six populations), because on primary feathers (including P1) the growth bars are barely visible. GBW are not visible on less than 5% of Ta6 feathers, while on approximately 15% of Ta5 few or no distinct growth bars could be distinguished. These individuals were excluded from the analyses that included GBW. We identified the proximal and distal limits of a feather segment including 3–11 bars, which were the number of bars that could be clearly seen in most individuals (for details, see [27]). The segment started from the first/second clearly visible band at the distal end of the feather and extended to cover the next bars towards the proximal end of the feather. We took a digital picture of each feather laying on a metric grid background and illuminated them with a spot light from a shallow angle to reveal the growth bars. Digital photographs were imported into ImageJ version 1.37 to measure the length of the segment with growth bars, and the GBW was expressed as the length of the segment divided by the number of growth bars included, as commonly done in ptilochronological studies [28]. Hence, large GBW indicates rapid feather growth. GBW increases from the proximal to the distal end of the feather (pers. obs.), and in case of non-random distribution of measurement positions between sexes and populations, this variation in band-width along the feather can affect the results. Therefore, we re-ran all models where GBW position (proximal, middle and distal) was included as a factor, but because the results remained unchanged, we only present the models without the position of measures. All measurements were performed by GO and LB without being aware of the tested hypothesis. Feather quality traits were highly repeatable with generally narrow confidence intervals, indicating that the traits can be measured with high accuracy (for all feather traits *R* > 0.7, randomly re-measured 15 individuals).

### Statistical Analyses

In the first set of analyses, we tested whether the measured traits (rachis width, feather length, barb density, barbule density, bending stiffness, GBW) of the Romanian samples differed among feather types (i.e. Ta5, Ta6 and P1) and between the sexes. Linear mixed-effects models were used, and for each feather trait we built a separate model that included the identity of individuals as a random factor, and sex and feather type as fixed factors. Because rachis width and barb density may correlate with feather length, barbule density with barb density, and bending stiffness with feather length and rachis width [23], we repeated the first set of analyses by including as continuous fixed effects those feather traits that covary with the focal response variable. To test the relationship between feather traits and GBW of tail feathers (Ta5 and Ta6), in a second set of analyses we used linear mixed-effects models of each feather trait, including bird identity as a random factor, sex and feather type as fixed factors and GBW as a continuous fixed term. In these models we choose to exclude any feather traits as a covariate, because none of these correlated with GBW (see Results).

In a third set of analyses we tested for population and sex differences in feather quality traits of Ta6 collected from six European barn swallow populations. For each feather trait we ran a separate linear model, including sex and population as explanatory factors. These analyses were then repeated while also including potentially confounding feather traits in the models (see above). To test the relationship between feather traits and GBW, in the fourth set of analyses we ran separate linear models of each feather trait including population and sex as a factors and GBW as a covariate.

All second order interactions were tested in each model set. We report minimal models, which were reached by removing the non-significant interactions, but the main effects were retained in every case no matter their *P*-value. All statistical analyses were conducted using the R statistical environment, version 3.1.2 [29]. Mixed models were constructed using the "lme4" package [30]. For the graphical presentation of the results, model estimates and corresponding standard errors were extracted from the models using the "effects" package [31].

## Results

### Difference among Feather Types

**Fig 1:**
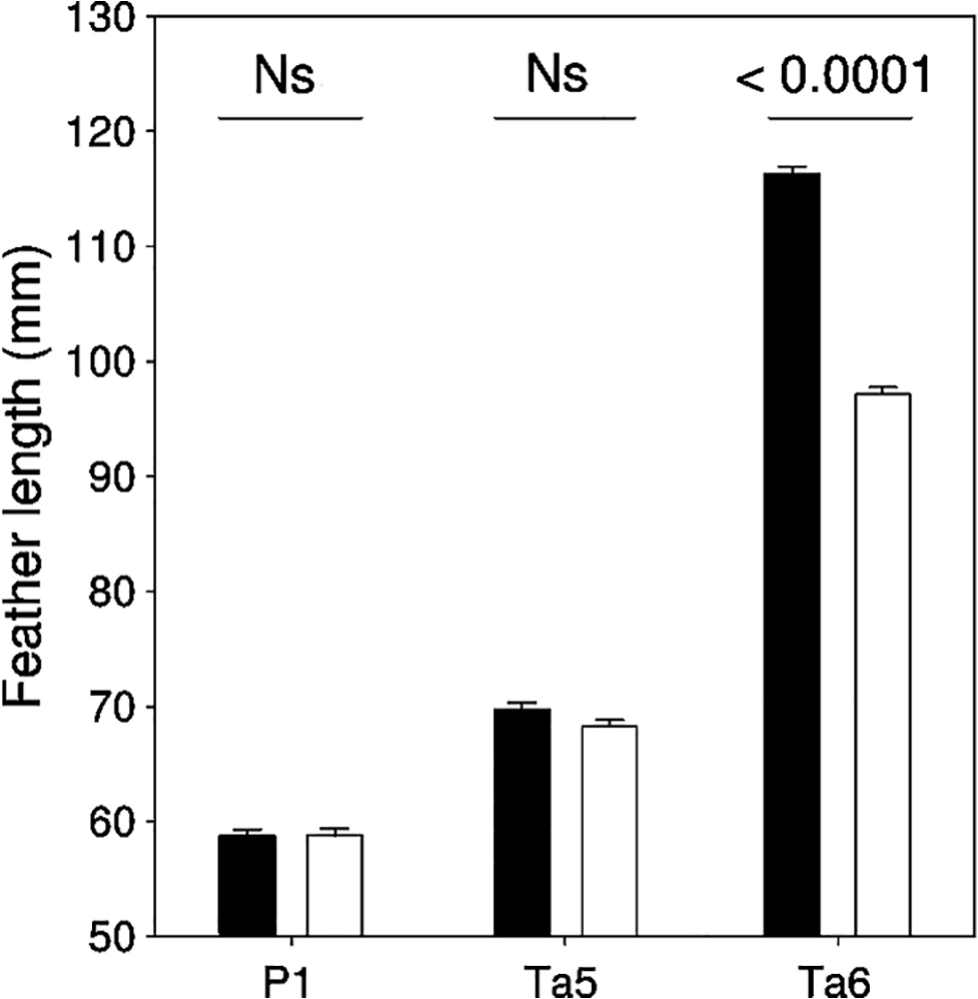
Variation in length of P1, Ta5 and Ta6 in male (black) and female (white) barn swallows from the Romanian population (mean + SE). Significance level for the difference between sexes for each feather types is shown above bars.

**Fig 2:**
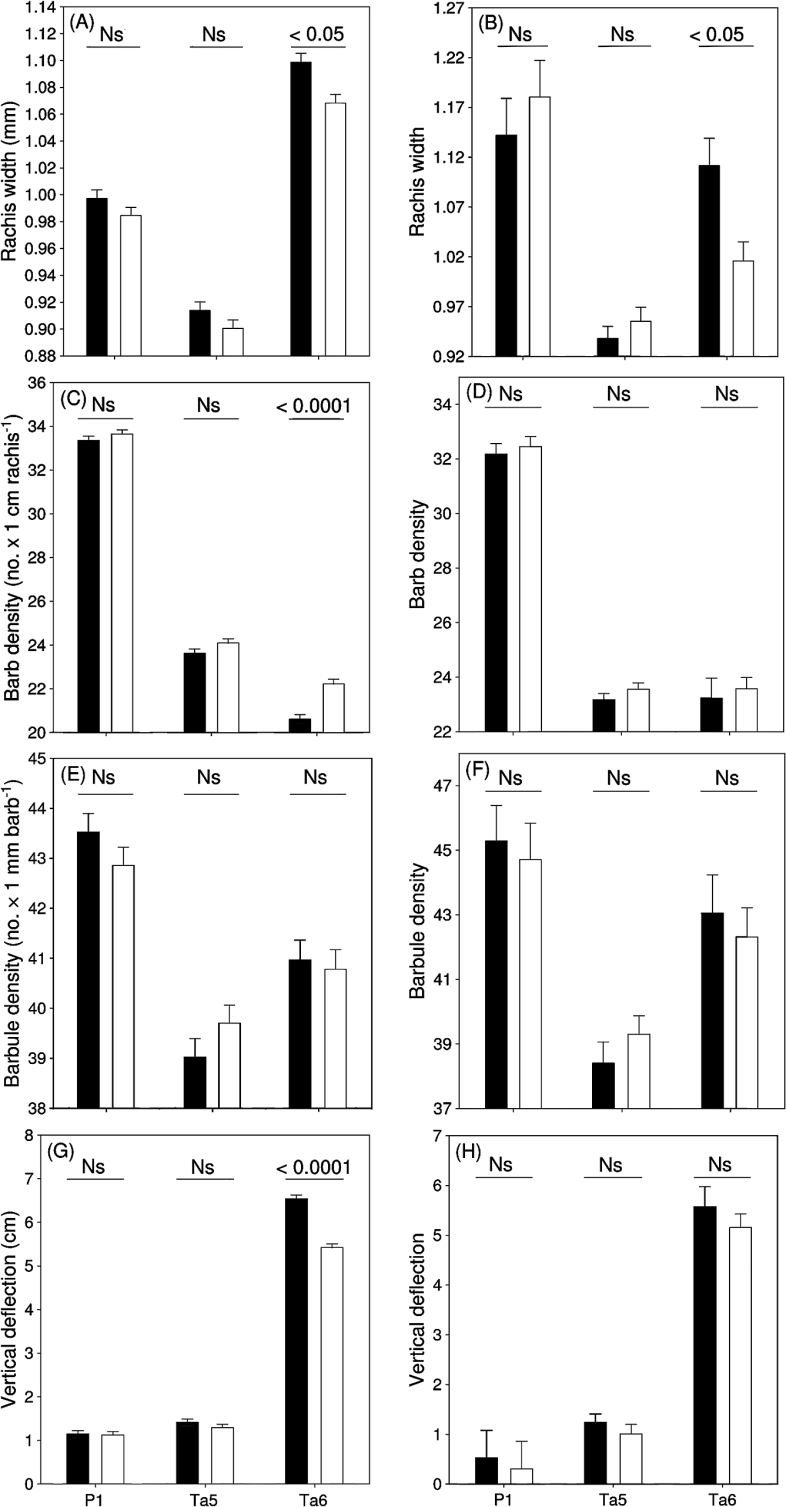
Variation in feather quality traits of P1, Ta5 and Ta6 in male (black) and female (white) barn swallows from the Romanian population (mean + SE). Figures in the left column are from the models without confounding variables included, while figures from the right column are from models with confounding variables included. For details, see Materials and methods. Significance level for the difference between sexes for each feather type is shown above bars.

Feather length differed significantly between feather types and sexes, and the significant sex × feather type interaction indicates that the difference between sexes was non-significant for P1 and Ta5, while Ta6 was significantly longer in males than in females (Table 1, Fig 1). Rachis diameter differed significantly between feather types and was the largest for Ta6, intermediate for P1 and the smallest for Ta5 (Table 1, Fig 2A). Rachis diameter was larger in males than in females for all feathers, as indicated by the significant effect of sex and the non-significant sex × feather type interaction (Table 1, Fig 2A). After accounting for the covariation between rachis width and feather length (*β* (SE) = 0.01 (0.002), *t* = 3.99, *P* = 0.0047), the difference between feather types remained significant (Table 1), although the significant difference between the sexes disappeared. The sex × feather type interaction became significant showing that residual rachis diameter of males was larger than that of females for Ta6, while no significant differences were observed for P1 and Ta5 (Table 1, Fig 2B). Barb density differed significantly between feather types, and was highest for P1, followed by Ta5 and Ta6 (Table 1, Fig 2C). Barb density was higher in females than in males for all feather types, and the difference between sexes was the largest in Ta6 as compared to P1 and Ta5, as indicated by the significant sex × feather type interaction (Table 1, Fig 2C). After including feather length in the model to control its significant and negative relationship with barb density (*β* (SE) = -0.07 (0.01), *t* = 5.69, *P* < 0.0001), the difference between feather types remained significant, with the highest value for P1 (Table 1, Fig 2D). However, the significant difference between sexes disappeared for all feather types, as indicated by the non-significant sex and sex × feather type interaction (Table 1, Fig 2D). Barbule density differed significantly between feather types, and was the highest for P1, followed by Ta6 and Ta5 (Table 1, Fig 2E). Barbule density was similar in the two sexes as indicated by the non-significant effect of sex and sex × feather type interaction (Table 1, Fig 2E). Including in the model barb density as a possible confounding variable of barbule density (*β* (SE) = -0.26 (0.15), *t* = 1.76, *P* = 0.50), the difference between feather types and sexes remained unchanged (Table 1, Fig 2F). Bending stiffness significantly differed between feather types, and was the lowest for Ta6, followed by Ta5 and P1 (Table 1, Fig 2G). Deflection of feathers was significantly higher in males than in females, and the significant sex × feather type interaction indicated that the difference between sexes increased from P1 and Ta5 to Ta6 (Table 1, Fig 2G). After including feather length and rachis width to control their significant and positive effect on deflection (feather length: *β* (SE) = 0.05 (0.04), *t* = 1.35, *P* < 0.0001; rachis width: *β* (SE) = 4.84 (2.45), *t* = 1.98, *P* = 0.0090), the difference between feather types remained significant (Table 1, Fig 1H). However, the difference between sexes decreased and turned to be similar for each feather type, as indicated by the non-significant sex and sex × feather type interaction (Table 1, Fig 1H).

**Table 1:** Linear mixed-effects models of variation in feather structure of wing (P1) and tail feathers (Ta5 and Ta6) between sexes of barn swallows from the Romanian population.

	Models without confounding factors included			Models with confounding factors included		
	df	*χ* ^2^	*P*	df	*χ* ^2^	*P*
Feather length						
Sex	1	117.56	< 0.0001	–		
Feather type	2	9550.42	< 0.0001	–		
Sex × feather type	2	429.92	< 0.0001	–		
Rachis width						
Sex	1	6.36	0.01	1	2.46	0.12
Feather type	2	1321.19	< 0.0001	2	642.56	< 0.0001
Feather length				1	7.99	0.0047
Sex × feather type				2	7.95	0.0188
Sex × feather length				1	7.08	0.0078
Feather type × feather length				2	22.28	< 0.0001
Barb density						
Sex	1	13.41	0.0003	1	2.42	0.1197
Feather type	2	5892.79	< 0.0001	2	2492.94	< 0.0001
Feather length				1	32.33	< 0.0001
Sex × feather type	2	17.93	0.0001			
Barbule density						
Sex	1	0.02	0.88	1	0.04	0.84
Feather type	2	126.50	< 0.0001	2	50.77	< 0.0001
Barb density				1	0.46	0.50
Sex × feather type				2	6.41	0.04
Feather type × barb density				2	7.10	0.03
Bending stiffness						
Sex	1	29.71	< 0.0001	1	4.88	0.028
Feather type	2	4824.16	< 0.0001	2	285.34	< 0.0001
Feather length				1	59.24	< 0.0001
Rachis width				1	6.82	0.0090
Sex × feather type	2	59.62	< 0.0001	–		
Sex × feather length				1	11.71	0.0006
Feather type × feather length				2	9.42	0.0090
Feather length × rachis width				1	8.07	0.0045

In the models only the significant interactions are presented.

### Feather Growth Rate and Feather Quality

**Fig 3:**
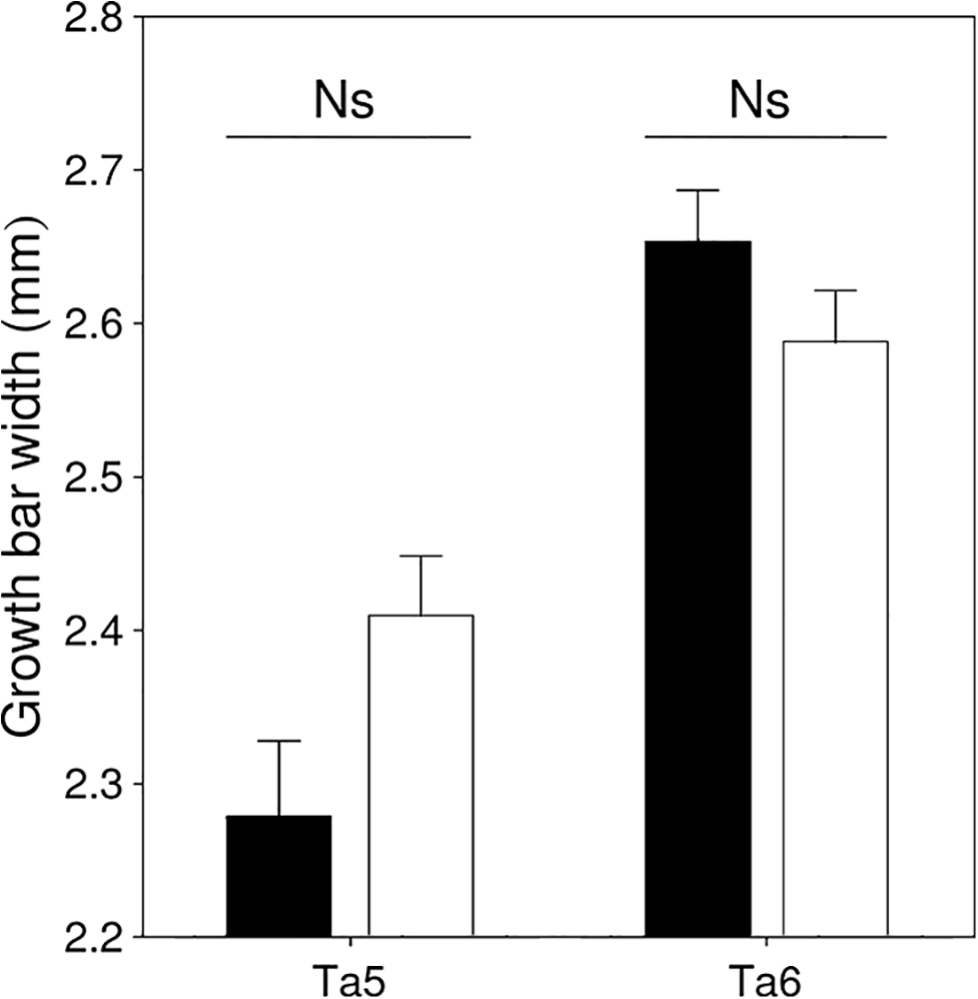
Variation in GBW of Ta5 and Ta6 in male (black) and female (white) barn swallows originating from the Romanian population (mean + SE). Significance level for the difference between sexes for each feather type is shown above bars.

We tested if the difference between sexes and feather types in feather quality is determined by variation in GBW. GBW was significantly larger for Ta6 than for Ta5 (Table 2, Fig 3). The significant sex × feather type interaction indicates that GBW of Ta5 was larger in females than in males, while the opposite was observed for Ta6 (Fig 3). None of the feather traits of Ta5 and Ta6 were predicted by GBW, as revealed by the non-significant effect of GBW in separate models where the effect of sex and feather type was controlled (Table 2).

**Table 2:** Linear mixed-effects models of the relationship between quality traits and GBW of tail feathers (Ta5 and Ta6) of male and female barn swallows from the Romanian population.

	df	*χ* ^2^	*P*
GBW			
Sex	1	0.03	0.87
Feather type	1	45.67	< 0.0001
Sex × feather type	1	6.23	0.01
Feather length			
Sex	1	153.06	< 0.0001
Feather type	1	1789.66	< 0.0001
GBW	1	0.83	0.36
Sex × feather type	1	153.11	< 0.0001
Rachis width			
Sex	1	7.23	0.007
Feather type	1	665.87	< 0.0001
GBW	1	0.14	0.71
Barb density			
Sex	1	31.93	< 0.0001
Feather type	1	119.27	< 0.0001
GBW	1	0.35	0.56
Barbule density			
Sex	1	0.00	0.95
Feather type	1	9.08	0.003
GBW	1	0.60	0.44
Bending stiffness			
Sex	1	35.07	< 0.0001
Feather type	1	948.18	< 0.0001
GBW	1	1.19	0.28
Sex × feather type	1	10.45	0.001

In the models only the statistically significant interactions are shown.

### Variation in Feather Traits among Populations

**Fig 4:**
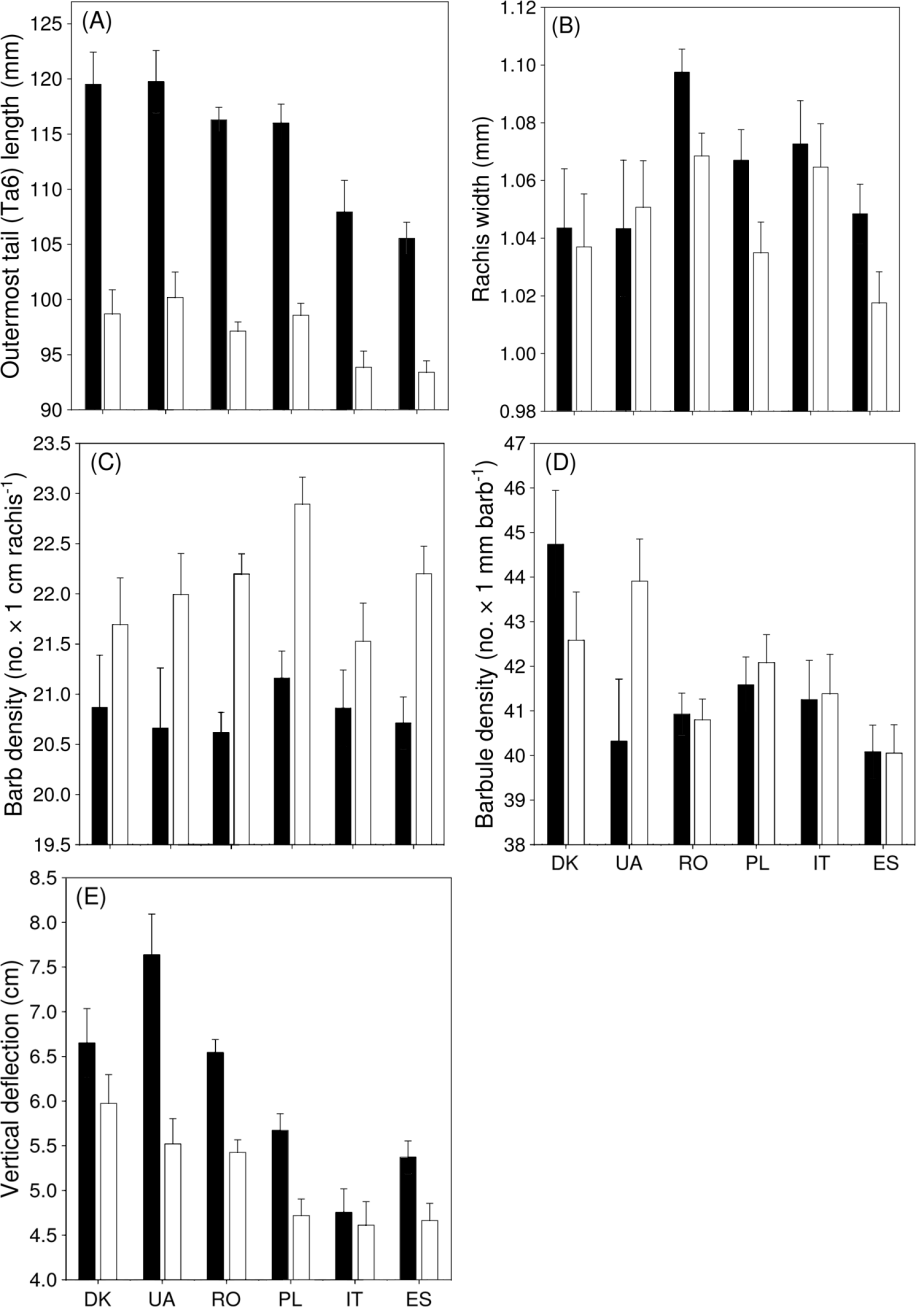
Variation in feather quality traits of Ta6 in male (black) and female (white) barn swallows from six European populations (DK—Denmark, UA—Ukraine, RO—Romania, PL—Poland, IT—Italy, ES—Spain) (mean + SE).

The length of Ta6 varied significantly among populations. Males had significantly longer outermost tail feathers than females (Table 3, Fig 4A). The significant interaction between population and sex indicated that sexual dimorphism in Ta6 length varied among populations. Rachis diameter was significantly larger in males than in females, and the non-significant sex × population interaction indicated that the difference between males and females in this trait was similar among populations (Table 3, Fig 4B). The significant variation in rachis width between populations was unrelated to sexual dimorphism in tail length, as change in this trait among populations was found not to be associated with sexual dimorphism in Ta6 (Fig 4B). None of the results changed after controlling for the significant and positive effect of feather length on rachis width (*β* (SE) = 0.16 x 10^−2^ (0.04 × 10^−2^), *t* = 3.67, *P* = 0.0003; Table 3). Barb density was significantly higher in females than in males, and the non-significant interaction between population and sex indicated that the sexual dimorphism in this trait was similar among populations (Table 3, Fig 4C). A near-significant difference in barb density was found between populations, and the change between populations was unrelated to sexual dimorphism in tail length (Table 3, Fig 4C). None of the results changed after controlling for the significant and negative effect of feather length on barb density (*β* (SE) = -0.06 (0.01), *t* = 5.54, *P* < 0.0001; Table 3). Barbule density was similar between sexes and differed significantly among populations (Table 3, Fig 4D). The change in barbule density among populations was unrelated to sexual dimorphism in tail length (Fig 4D). None of the results changed after controlling for the possible confounding effect of barb density on barbule density (Table 3). Bending stiffness was consistently and significantly larger in females than in males, as indicated by the significant sex effect and the non-significant sex × population interaction (Table 3, Fig 4E). Population significantly explained variation in bending stiffness, although the change was unrelated to sexual dimorphism in tail length (Fig 4E). None of the results was affected by the significant and positive effect of feather length and rachis width on deflection (feather length: *β* (SE) = 0.05 (0.01), *t* = 6.22, *P* < 0.0001; rachis width: *β* (SE) = 3.11 (1.02), *t* = 3.04, *P* = 0.003; Table 3).

**Table 3:** Linear models of the population and sex differences in quality traits and growth of the outermost tail feather (Ta6) of barn swallows.

	Models without confounding factors included			Models with confounding factors included		
	df	*F*	*P*	df	*F*	*P*
Feather length						
Sex	1	352.07	< 0.0001	–		
Population	5	12.96	< 0.0001	–		
Sex × population	5	2.24	0.05	–		
Rachis width						
Sex	1	13.05	< 0.0001	1	13.62	< 0.0001
Population	5	6.86	< 0.0001	5	7.16	< 0.0001
Feather length				1	13.47	< 0.0001
Barb density						
Sex	1	69.55	< 0.0001	1	76.72	< 0.0001
Population	5	2.07	0.07	5	2.29	0.05
Feather length				1	30.69	< 0.0001
Barbule density						
Sex	1	0.42	0.52	1	0.42	0.52
Population	5	4.39	0.0007	5	4.39	0.0007
Barb density				1	0.80	0.37
Bending stiffness						
Sex	1	47.61	< 0.0001	1	54.46	< 0.0001
Population	5	16.30	< 0.0001	5	18.65	< 0.0001
Feather length				1	32.86	< 0.0001
Rachis width				1	9.27	0.003

### Covariation between Feather Quality and Feather Growth Rate among Populations

**Fig 5:**
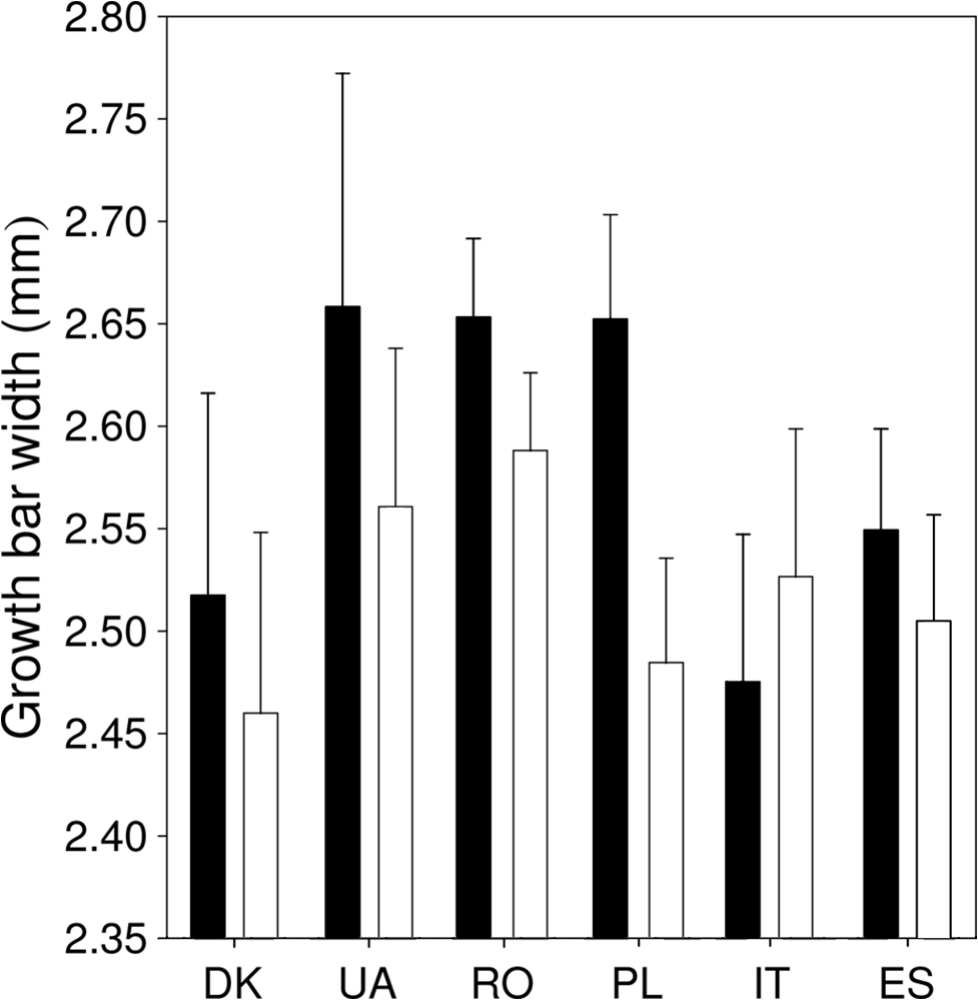
Variation in GBW of Ta6 in male (black) and female (white) barn swallows from six European populations (DK—Denmark, UA—Ukraine, RO—Romania, PL—Poland, IT—Italy, ES—Spain) (mean + SE).

We tested if the difference between sexes and populations in feather quality traits of Ta6 could be explained by GBW. We included GBW as an explanatory variable in separate models, while controlling for the effect of sex and population. GBW of Ta6 was significantly larger in females than in males, while population had no effect on GBW (Table 4, Fig 5). Feather length, rachis width, barbule density and bending stiffness were unaffected by GBW (Table 4). However, GBW was significantly and negatively related to barb density: feathers that grew fast had low density of barbs in the vane (*β* (SE) = -1.15 (0.30), *t* = 3.77, *P* = 0.0002; Table 4).

**Table 4:** Linear models of the relationship between quality traits and GBW of the outermost tail feather (Ta6) of male and female barn swallows from six European populations.

	df	*F*	*P*
GBW			
Sex	1	4.58	0.03
Population	5	1.74	0.13
Feather length			
Sex	1	344.08	< 0.0001
Population	5	12.66	< 0.0001
GBW	1	0.52	0.47
Rachis width			
Sex	1	13.10	0.0003
Population	5	6.89	< 0.0001
GBW	1	2.13	0.15
Barb density			
Sex	1	72.74	< 0.0001
Population	5	2.17	0.06
GBW	1	14.23	0.0002
Barbule density			
Sex	1	0.42	0.52
Population	5	4.37	0.0007
GBW	1	0.00	0.98
Bending stiffness			
Sex	1	48.74	< 0.0001
Population	5	16.69	< 0.0001
GBW	1	0.94	0.33
Sex × population	5	2.33	0.04

In the models only the statistically significant interactions are shown.

## Discussion

### Feather Trait Variation among Sexes and Feather Types

The higher density of the vane (as defined by the number of barbs and barbules per unit of length) increases the resistance to airflow and the capacity to produce high lift-to-drag ratio [32,33]. Feathers of flying birds have to withstand aerodynamic forces during flight [34–37], and because these forces are higher on primaries than on rectrices [8–12], we expected difference in structural properties of the vane. Our results support this prediction, as indicated by high barb and barbule density of P1 as compared to tail feathers (Fig 2C–2F).

Sexes were similar in barb and barbule density for P1 and Ta5, as expected, owing to the absence of sexual dimorphism in length of these feathers (Fig 1; see [38]). In contrast, barb density of Ta6 was significantly lower in males as compared to females, reflecting the cost of elongation of this trait [16,39,40]. In fact, the significant negative relationship between barb density and feather length, and the absence of difference in relative barb density of Ta6 between the sexes after controlling for tail length indicates that reduced barb density in males is related to the high inter-barb distance of longer tails. The existence of a cost of elongation of Ta6 is further supported by reduced barb density of this feather compared to the shorter Ta5 (Fig 2C). As feather length correlates negatively with the density of barbs, the difference in barb density between Ta5 and Ta6 turned non-significant after controlling for feather length (Fig 2D). This result confirms again that elongation of Ta6 in the barn swallow entails reduction of barb density of the vane. Barbule density, on the other hand, was higher in Ta6 than in Ta5, and did not differ between the sexes for all feather types, even after the potentially confounding effect of barb density was controlled (Fig 2E and 2F). This finding suggests that the elongation of Ta6 evolved without compromising barbule density of the vane. That absence of difference in barbule density of Ta6 between the sexes can be explained by the fact that the feather elongation (length) affects only barb density, but not barbule density of the feather [23]. The high barbule density of Ta6 compared to Ta5 may have evolved to compensate for reduced barb density in the longer Ta6.

We found that rachis width, which determines the flexibility of the feather and hence its response to aerodynamic forces and bending moments in flight [37], was significantly larger in P1 than in Ta5 (Fig 2A and 2B). The difference in rachis width between P1 and Ta5 was reflected in bending stiffness, as indicated by the higher stiffness of wing relative to tail feathers (Fig 2G and 2H). The high resistance to bending of P1 can be explained by the importance of wing feathers in resistance against the high aerodynamic forces during flight [4]. The high relative rachis width of Ta6 relative to Ta5 (Fig 2A and 2B) suggests that the increased aerodynamic forces at the leading edge of the tail may be responsible for the large investment in rachis strength of this feather even if this might be constrained by elongation (see [15] for the function of tail feathers in flight). Our results show that rachis width of Ta6 is affected by sexual selection on length of this trait, as indicated by the significantly larger rachis width of males than females, even after the difference in feather length between the sexes was controlled (Fig 2A and 2B). However, the difference between sexes in bending stiffness of Ta6 is solely determined by sexual dimorphism in length and rachis width, as indicated by the absence of difference in bending stiffness in Ta6 after controlling for length and rachis width (Fig 2H). In contrast, even after controlling for the difference among feather types in length and rachis width, the flexibility of Ta6 was the highest (Fig 2H), which shows that other traits, like the keratin microstructure of the rachis (e.g. second moment of rachis’ cross-sectional area), are responsible for the difference in bending stiffness between feather types (see [4]).

### Among Population Variation in Feather Traits

The difference in feather quality traits between populations changed inconsistently even after populations were arranged according to latitude or to assumed migration distance between breeding and wintering sites (from the longest to the shortest migration distance: Denmark, Poland, Ukraine, Romania, Italy, Spain; results not shown. For wintering sites of different barn swallow populations, see [16,41,42]). If sexual selection on tail length was the only selective force affecting this trait, we would expect an increase in rachis width and bending stiffness, and a decrease in barb density among populations arranged according to sexual dimorphism in tail length. However, our results show that all feather quality traits, except bending stiffness, varied mostly independent of sexual dimorphism in Ta6 (Fig 4A–4E). Hence, the quality of tail feathers differed among populations, but the investment in this trait is, besides sexual selection, affected by other concomitant natural selective pressures [16]. On the other hand, consistency in dimorphism in feather quality traits among populations shows that the elongation of Ta6 in male European barn swallows has affected the structural component of this trait similarly among populations.

### Covariation between Feather Growth Rate and Feather Quality

Feather growth rate is an important determinant of feather quality and fitness because fast growth may impair the quality of flight feathers, which may ultimately affect flight performance, breeding success and survival [21,26–28,43]. On the other hand, fast growth may allow the early start of spring migration in the winter-molting barn swallow, which may determine arrival date and hence the number of breeding events [44]. In fact, Saino et al. [27] show that growth rate of the 4^th^ tail feather (counting outwards) at the African wintering grounds significantly and positively predicted body condition and the number of fledglings produced during the next breeding season in older males. Our results show that Ta5 and Ta6 differ in GBW and the difference among feather types vary between males and females. The significantly faster growth rate of Ta6 than that of Ta5 (Fig 3) can be explained by the long time needed to fulfill the growth of a longer feather, which is even longer in males than in females. Therefore, one may expect that costs in terms of time and energy might constrain the quality of Ta6 more than that of Ta5, and more in males than in females. The significant negative correlation between GBW of Ta6 and barb density measured in birds from different populations (Table 4) support this conclusion. However, other feather quality traits were not related to GBW, measured on Ta5 and Ta6 of the Romanian barn swallows or on Ta6 from the six European populations, indicating that growth rate has generally little effect on the quality of tail feathers. Instead, GBW may indicate the general condition of the individual during molt [27]. The absence of a significant difference in GBW among populations supports the conclusion that population differences in sexual dimorphism and hence the strength of sexual selection have no effect on the growth of Ta6, while local factors during molt are probably more important in determining the growth rate of this feather.
